# Geometric Analysis to Determine Kinking and Shortening of Bridging Stents After Branched Endovascular Aortic Repair

**DOI:** 10.1007/s00270-021-02773-w

**Published:** 2021-02-19

**Authors:** Alice Finotello, Giovanni Spinella, Giulia Notini, Domenico Palombo, Giovanni Pratesi, Simone Mambrini, Ferdinando Auricchio, Michele Conti, Bianca Pane

**Affiliations:** 1 Department of Surgical Sciences and Integrated Diagnostic, University of Genoa, Genoa, Italy; 2 Vascular and Endovascular Surgery Unit, Ospedale Policlinico San Martino, University of Genoa, Largo Rosanna Benzi, 10, 16132 Genoa, Italy; 3 Department of Civil Engineering and Architecture, University of Pavia, Pavia, Italy

**Keywords:** Bridging stent, B-EVAR, Self-expanding stent-graft, Balloon-expanding stent-graft, Thoraco-abdominal aortic aneurysm, Geometric analysis

## Abstract

**Purpose:**

To evaluate bridging stent geometry in patients who underwent branched endovascular aortic repair (B-EVAR) and to correlate the outcomes with intrinsic bridging stent characteristics aiming to identify the stent(s) that guarantees the best performance.

**Methods:**

Pre-operative and post-operative computed tomography images of all patients undergoing B-EVAR between September 2016 and April 2019 were retrospectively analyzed. Following geometrical features were measured: target vessel take-off angle (TOA); longitudinal stent shortening; shape index (SI), intended as ratio between minimum and maximum diameter of the lumen cross sections, averaged on three segments: zone 1 (proximal stented zone), zone 2 (intermediate), and zone 3 (distal).

**Results:**

Thirty-eight branches (8 right (RRA) and 8 left renal arteries (LRA), 11 superior mesenteric arteries (SMA), 11 celiac trunks (CTR)) were treated. Fluency (Bard Peripheral Vascular), COVERA (Bard Peripheral Vascular), and VBX (WLGore&Assoc) stent-grafts were implanted in 10, 12, and 16 branches, respectively. Pre-operative TOA was more acute in RRA and LRA when compared to CTR and SMA, and straightened in post-operative configuration (109.86 ± 28.65° to 150.27 ± 21.0°; *P* < 0.001). Comparable values of SI among the stent types were found in zone 1 (*P* = 0.08), whereas higher SI in VBX group was detected in zones 2 (*P* < 0.001) and 3 (*P* < 0.001). The VBX group was also the most affected by stent shortening (11.12 ± 5.65%; *P* = 0.001).

**Conclusion:**

Our early experience showed that the VBX stent offers greater stent circularity than the other devices even if a greater shortening has been observed drawing attention with regards to the decision of the nominal stent length.

## Introduction

In the recent years, with the introduction of fenestrated endovascular aneurysm repair (F-EVAR) and more recently branched stent-graft (B-EVAR), endovascular repair has become a valid solution in the treatment of thoracoabdominal aortic aneurysms (TAAA) for those patients not suitable for open surgery [1–3]. The choice between the two types of configurations (F-EVAR *vs* B-EVAR) is usually made on the basis of the target vessel aortic diameter as well as orientation of renal arteries [4, 5]. Moreover, the availability of off-the-shelf devices for B-EVAR favors the choice toward this type of intervention in case of emergency settings.

Despite many aspects having been investigated concerning F-EVAR, few studies have been reported with respect to B-EVAR to date. Comparative studies have recently been published demonstrating that patency for the side branches is greater when F-EVAR is adopted, while the reoperation rates are lower in cases where branches were used [6]. Occlusion causes in B-EVAR could be related to the greater length of the branch stents if compared to the fenestrated ones and to the angles that are induced along the stent in the case of B-EVAR [7]. Patency outcomes at medium and long-term follow-up referred to the new generation of stents are still few. The multicentric experience reported by Silingardi et el. [8] with off-the-shelf multibranched endografts and bridging for visceral and renal vessel performed by balloon-expandable and/or self-expanding covered stents showed at median follow-up of 18 months, 3 of 73 cases of branch occlusion and 5 reinterventions.

Clinical outcomes at medium term follow-up with Covera Plus stent bridge have been recently reported by Gennai et al. [9]. Over 12 months follow-up, they reported 100% patency and no reintervention.

In particular, available B-EVAR studies refer to two macro-categories of bridging covered stents, i.e., balloon-expandable and self-expandable devices. Balloon-expandable stents have a higher radial stiffness, lower flexibility and they can crush and deform due to extrinsic compression. On the contrary, self-expandable devices are more flexible, allow radial compliance and are conceived to better accommodate tortuous anatomies [10]. These two types of stents have also been adopted in combination to achieve as close to ideal desired characteristics as possible [11]. In fact, the quest for an ideal bridging stent that supports different characteristics along its length and guarantees a stable anchorage to the main body, while also ensuring excellent flexibility in the intermediate area and avoiding kinks in the most distal portion, has not to date had satisfactory results [12].

Our hypothesis is that radial stiffness, which is the main characteristic of balloon-expandable devices rather than radial compliance and conformability ensured by self-expandable devices, is a key feature in the treatment of renal and visceral vessels in order to limit complications during follow-up.

In light of this, carrying out an image-based geometric analysis as already performed in other vascular districts [13] to investigate the behavior of the already commercially available devices assumes great importance. However, to our knowledge, this has never been done in the context of bridging stents. Accordingly, the goal of our study is to evaluate bridging stent geometry in patients who underwent B-EVAR and to correlate the outcomes with intrinsic bridging stent characteristics aiming to identify the stent(s) that guarantees the best performance and additionally provide useful indications for the realization of a stent considered as ideal.

## Methods

### Study Cohort

A single-center retrospective study on patients who had undergone B-EVAR of TAAA at our center between September 2016 and August 2019 was conducted.

Inclusion criteria were: pre-operative and follow-up computed tomography angiography (CTA) scans, degenerative atherosclerotic TAAA, chronic type B aortic dissection TAAA, and B-EVAR configuration. Exclusion criteria were F-EVAR stent-graft configuration and patients with unavailable contrast-enhanced pre- or post-operative CTA scans.

### Devices and Procedure

Two types of stent-graft configurations were used: an “off-the-shelf” or a “custom-made” device. The off-the-shelf T-branch multi-branched endograft device (Cook Medical, Bloomington, IN, USA) is a premade stent-graft in a fixed configuration consisting in a tapered main body provided by four downward cylindrical cuffs and mounted on a 22F delivery system. Conversely, the custom-made device is a commercially available patient-tailored device provided by the manufacturer Cook Medical and based on the Zenith platform with two branches (celiac trunk and superior mesenteric artery) and two fenestrations (renal arteries). The manufacturing time extends up 12 weeks limiting their use in elective treatment. When the latter configuration was used, only the bridging stents for the branches were included in the analysis.

In our center, three different types of bridging stents are routinely adopted in B-EVAR cases: Fluency (Bard Peripheral Vascular, Tempe, AZ), Covera (Bard Peripheral Vascular, Tempe, AZ), and Viabahn VBX (WLGore & Assoc, Flagstaff, AZ). For a single patient, always the same type of bridging stent was implanted in one vessel, if additional stents were required.

In the cases of the positioning of a self-expanding stent (Fluency and Covera), the choice of stent diameter was made considering an oversizing of 1 mm from the nominal diameter of the target vessel. When balloon-expandable devices were adopted (Viabahn VBX), a 5– 7 mm balloon-expandable stent was positioned inside the renal arteries according to their nominal diameter without the need of oversizing. When positioning inside the celiac trunk and the superior mesenteric artery if target vessel diameter was superior to 8 mm, we opted for a Viabahn VBX 8L stent which could be oversized up to 13 mm.

### Image Acquisition and Processing

CTA images were analyzed and segmented to extract three-dimensional surface models of branched vessels and stents. All CTAs were acquired with the use of a 64 multidetector-row CTA unit Optima 660 (General Electric, Boston, Massachusset, USA) or a 64 multidetector-row CTA unit Somatom Definition Flash (Siemens, Erlagen, Germany). Slice thickness and pixel spacing were in the range 0.5–1.0 mm and 0.55–0.9, respectively. Both pre- and post-operative CTAs were acquired during inspiratory breath holds.

All morphological quantifications and geometric analysis were performed using Endosize® software (Therenva, France) and Vascular Modeling ToolKit (VMTK) libraries.

### Geometric Analysis

Different parameters were evaluated on pre-operative and post-operative CTA scans; all measurements were taken along the centerline.

**Fig. 1:**
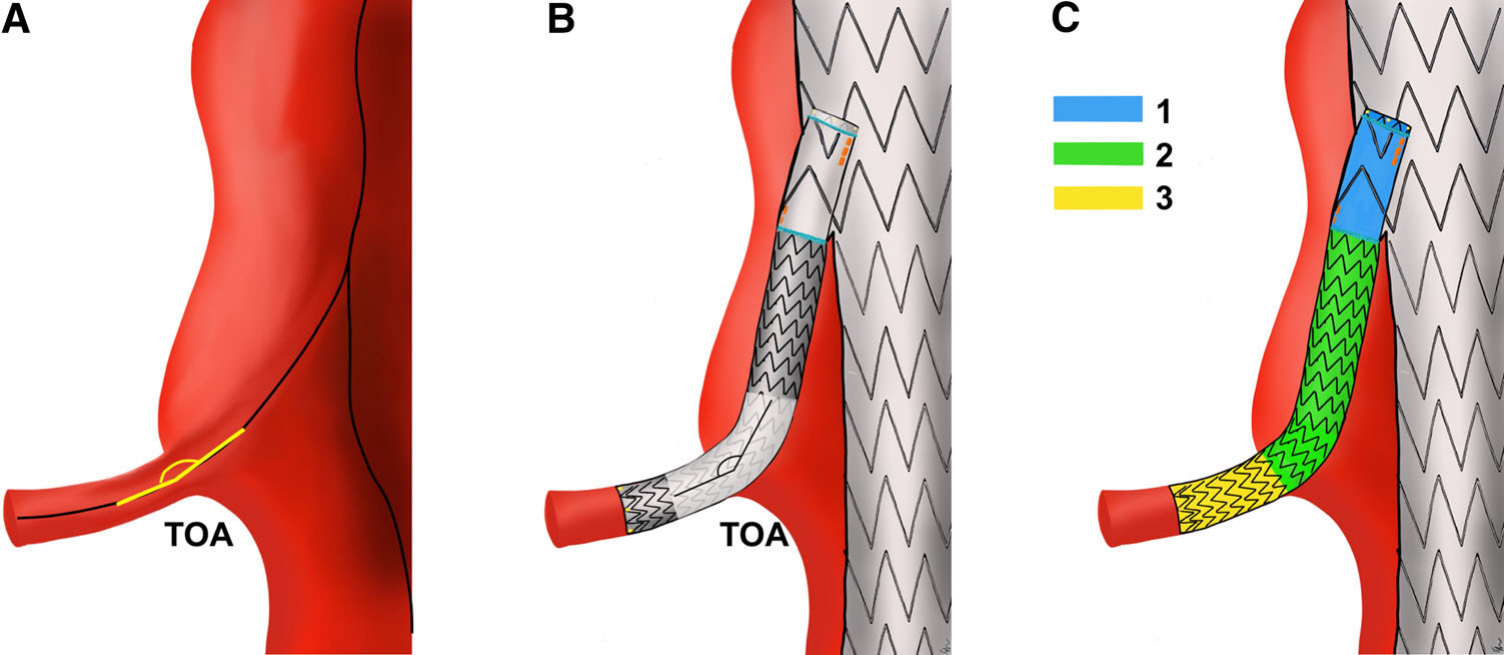
Schematic representation of computed parameters on CTA. **A** Pre-operative take-off angle (TOA); **B** post-operative TOA; **C** post-operative stent subdivision into zone 1 (light blue), zone 2 (green), and zone 3 (yellow) to compute centerline lengths and sections shape index

Pre-operative CTA examination included the measurement of the external wall aneurysm diameter and the aortic lumen diameter at target vessel origin, target vessel mean diameter, and target vessel take-off angle (TOA). The TOA was conceived as the target vessel origin angle from the aorta measured on the centerline path (see Fig. 1A); it was measured in degree, and values ranged from 0° (most acute angle) to 180° (straight angle).

Post-operative CTA geometric analysis was carried out to compute the subsequent measurements. Target vessel TOA angle on the stented artery was computed and compared to the corresponding pre-operative one (Fig. 1B).

As depicted in Fig. 1C, the stent was subdivided into three segments along its length: zone 1 from the stent origin to end of the main body branch; zone 2, from the end of the main body branch to the origin of the native vessel; zone 3, from the native vessel origin to the end of the stent.

After computing the centerline of each vessel, we analyze the vessel by generating sections, at given distances, perpendicular to the centerline. Then, for each section, we computed a Shape Index (SI) value which is a measure of the section eccentricity as the ratio between the sections’ minimum and the maximum diameter, according to Finotello et al. [14]. SI ranges from 0 to 1 where 1 signifies a completely circular shape. Stent length along the centerline and section SI were computed both taking into account the whole stent and the three segments.

Longitudinal stent shortening was computed as the percentage difference between the nominal stent length and the actual stent length measured along the post-operative centerline. This measurement was computed only for single stent implantations.

Although most of the operations performed, from lumen segmentation to centerline extraction, are based on semi-automatic scripts which minimize users’ interaction reducing user-related errors, measurement extraction could potentially lead to slight measurement differences. For this reason, two independent skilled operators performed segmentation of CTAs images and subsequent measurements extraction and one operator conducted it twice.

### Clinical Outcome Measurements

Technical success defined as successful main endograft and bridging stent implantations, spinal cord ischemia (SCI) classified as transient or permanent, and early 30-day mortality were assessed as early outcomes. Survival, freedom from reinterventions, bridging stent-patency and bridging instability were evaluated during follow-up.

### Statistical Analysis

Categorical variables were listed as numbers and percentages. Continuous variables were expressed as mean ± standard deviation. *T*-student test and one-way Anova test were adopted. Univariate correlations were examined using Pearson’s correlation coefficient. Intraobserver and interobserver variability in measurements were assessed in all patients with the intraclass correlation coefficient (ICC). The statistical analyses were performed with the MATLAB R2019b—Statistics and Machine Learning Toolbox-software (The MathWorks, Inc, Natick, MA).

## Results

### Patient Demographics

During the enrollment period, 16 patients underwent endovascular treatment for TAAAs. In accordance with the inclusion and exclusion criteria, five patients were excluded from the study due to the following reasons: four patients were treated for fenestrated design, and one case because different bridging stents were used.

In total, 38 vessels were treated and analyzed: 11 celiac trunks (CTR), 11 superior mesenteric arteries (SMA), 8 left (LRA), and 8 right renal arteries (RRA). Fluency, COVERA, and VBX stent-grafts were implanted in 10, 12, and 16 branches, respectively. Patient characteristics and risk factors are listed in Table 1. All patients underwent both pre- and post-operative CTAs with the post-operative imaging performed at a median of 3.0 days (range, 1–729 days).

**Table 1:** Patients’ demographics

Patients	11
Sex; male/female	10/1
Age; years	77
*Risk factors*	
Dislipidemia; n	3
Hypertension; n	9
Coronary artery disease; n	6
Diabetes mellitus; n	1
COPD; n	6
Renal Disease; n	3
Prior aortic repair; n	5
*Main device*	
Custom-made; n (bridging stents adopted)	3 (2 Fluency, 2 Covera, 6 VBX)
Off-the-shelf; n (bridging stents adopted)	8 (8 Fluency, 11 Covera, 10 VBX)
Vessels; total n	38
CTR; n (bridging stents adopted)	11 (3 Fluency, 2 Covera, 6 VBX)
SMA; n (bridging stents adopted)	11 (3 Fluency, 4 Covera, 4 VBX)
LRA; n ((bridging stents adopted)	8 (2 Fluency, 3 Covera, 3 VBX)
RRA; n (bridging stents adopted)	8 (2 Fluency, 3 Covera, 3 VBX)
*Stent types*	
Fluency; n (vessels)	10 (3 CTR, 3 SMA, 2 LRA, 2 RRA)
Covera; n (vessels)	12 (2 CTR, 4 SMA, 3 LRA, 3 RRA)
VBX; n (vessels)	16 (6 CTR, 4 SMA, 3 LRA, 3 RRA)

*CTR* celiac trunk; *SMA* superior mesenteric artery; *LRA* left renal artery; *RRA* right renal artery

Single stent was implanted in 34 vessels, whereas in 4 cases two partially overlapped devices were required (2 cases VBX, 1 Covera, and 1 Fluency). Mean overlap was quantified equal to 42.7 ± 6.8 mm. In the two VBX cases, extension was required for insufficient sealing into the target vessel, in the other two cases an additional self-expanding device was added to overcome loss of sealing between the bridging stent and main branch cuff.

### Clinical Outcomes

No 30-day mortality was observed. Transient SCI was observed in two cases and resolved after active cerebrospinal fluid drainage. No permanent SCI was observed. The average follow-up was 10.5 months. There was no compression of the celiac trunk exerted by median arcuate ligament of the diaphragm. During the follow-up, we observed three stent occlusions in two patients. In particular, in one patient bilateral renal stent occlusion (Fluency) was observed 14 months after treatment and 1 patient experienced left renal stent occlusion (VBX) 40 days after treatment. In both cases, stented renal arteries thrombized internally without any detection of stent kinking. During follow-up, one patient underwent relining of a CTR branch treated with VBX due to inadequate landing zone in the target vessel.

### Geometric Analysis

Outcomes of pre-operative geometric analysis comparing different target vessels were reported in Table 2. Target vessel diameter was 7.0 ± 1.1 mm for the total group of patients, being significantly lower in renal arteries (*P* = 0.04). Also nominal bridging stent diameter for LRA and RRA was inferior in comparison to CTR and SMA (*P* = 0.05).

**Table 2:** Pre-operative geometrical evaluations

	Total	CTR	SMA	LRA	RRA	*P*-value
Target vessel diameter, mm	7.0 ± 1.1	8.1 ± 0.6	7.8 ± 1.1	6.1 ± 1.1	5.9 ± 1.1	*P*-value = 0.04*
Nominal stent diameter, mm	7.6 ± 1.3	8.5 ± 0.9	8.7 ± 0.7	6.4 ± 0.7	6.4 ± 0.7	*P*-value = 0.05*
Pre-operative TOA, °	109.86 ± 28.65	117.5 ± 28.4	115.8 ± 28	104.5 ± 28.9	97.3 ± 29.6	*P*-value = 0.4
Aneurysm diameter (TOA level), mm	42.56 ± 11.88	50.9 ± 10.2	45.8 ± 13.9	34.7 ± 5.9	35.5 ± 6.4	*P*-value = 0.003***
Lumen diameter (TOA level), mm	32.17 ± 6.27	36.4 ± 6.1	34.3 ± 6.9	27.3 ± 2.5	28.5 ± 2.3	*P*-value = 0.004***

*TOA* take-off angle. Statistical significance: “*” for 0.05; “**” for 0.005, and “***” for < 0.005

Pre-operative CTA scans showed TOA for the renal arteries (LRA 104.5 ± 28.9°; RRA 97.3 ± 29.6°) was more acute when compared to celiac trunks (117.5 ± 28.4°) and superior mesenteric arteries (115.8 ± 28°), although statistical significance was not reached (*P* = 0.4).

The aneurysm external wall diameter was found to be smaller at the level of the pararenal aorta (LRA 34.7 ± 5.9 mm; RRA 35.5 ± 6.4 mm) than at suprarenal aorta level (CTR 50.9 ± 10.2 mm; SMA 45.8 ± 13.9 mm) (*P* = 0.003). Similarly, aortic lumen diameter was smaller in correspondence to the renal arteries (LRA 27.3 ± 2.5 mm; RRA 28.5 ± 2.3 mm) than at level of CTR (36.4 ± 6.1 mm) and SMA (34.3 ± 6.9 mm) (*P* = 0.004). No significant differences were found on pre-operative measurements if grouping by different bridging stent types.

Results of follow-up geometric analysis taking into consideration both the total group of patient and the bridging stent types and the target vessels are reported in Table 3.

**Table 3:** Post-operative geometrical evaluations

	Total	Fluency	Covera	VBX	*P*-value	CTR	SMA	LRA	RRA	*P*-value
TOA, °	150.27 ± 21	156.75 ± 13.74	158.2 ± 11.7	140.6 ± 26.2	*P* = 0.05*	154.1 ± 15.8	156.5 ± 21.6	148.4 ± 22.5	140.0 ± 23.4	*P* = 0.3
Zone 1 —length, mm	18.11 ± 0.93	17.71 ± 0.63	18.19 ± 0.92	18.37 ± 1.02	*P* = 0.4	18.5 ± 1.3	18.2 ± 0.9	17.7 ± 0.7	18.0 ± 0.04	*P* = 0.1
Zone 1 —SI, -	0.89 ± 0.04	0.88 ± 0.03	0.87 ± 0.04	0.91 ± 0.03	*P* = 0.08	0.89 ± 0.04	0.89 ± 0.06	0.87 ± 0.06	0.88 ± 0.04	*P* = 0.3
Zone 2 –length, mm	30.97 ± 10.0	27.7 ± 8.6	32.33 ± 12.4	32 ± 9.0	*P* = 0.4	32.8 ± 11.7	31.2 ± 7.3	28.6 ± 6.2	30.5 ± 14.2	*P* = 0.08
Zone 2 —SI, -	0.88 ± 0.06	0.81 ± 0.06	0.89 ± 0.03	0.92 ± 0.02	*P* < 0.001***	0.88 ± 0.07	0.88 ± 0.06	0.88 ± 0.05	0.88 ± 0.05	*P* = 0.5
Zone 3 —length, mm	21.84 ± 10.27	23.5 ± 7.26	22.58 ± 11.4	19.25 ± 11.3	*P* = 0.08	21.1 ± 8.8	19.2 ± 8.0	22.0 ± 11.9	24.4 ± 13.3	*P* = 0.07
Zone 3 —SI, -	0.88 ± 0.06	0.81 ± 0.05	0.88 ± 0.05	0.93 ± 0.02	*P* < 0.001***	0.88 ± 0.06	0.89 ± 0.06	0.87 ± 0.06	0.89 ± 0.03	*P* = 0.1
Nominal stent length, mm	70.26 ± 11.3	66.67 ± 10.0	72.73 ± 13.5	70.64 ± 10.5	*P* = 0.5	68.7 ± 11.0	69.6 ± 10.3	69.6 ± 10.6	74.1 ± 12.2	*P* = 0.1
Stent total length, mm	65.61 ± 11.3	63.00 ± 6.9	71.00 ± 12.6	63.08 ± 11.8	*P* = 0.4	63.7 ± 11.8	66.1 ± 8.8	65.6 ± 11.2	68.8 ± 14.3	*P* = 0.2
Stent shortening, %	− 6.58 ± 6.42	− 4.86 ± 4.63	− 2.23 ± 2.18	− 11.12 ± 5.65	*P* = 0.001***	− 7.4 ± 5.8	− 4.7 ± 4.3	− 5.9 ± 5.3	− 6.8 ± 6.6	*P* = 0.09

*TOA* take-off angle; *SI* shape index. Statistical significance: “*” for 0.05; “**” for 0.005, and “***” for < 0.005

As concern target vessel subdivision, the mean post-operative TOA was less acute than the pre-operative one (109.86 ± 28.65° to 150.27 ± 21°; *P* < 0.001). Similar to the pre-operative data, the post-operative TOA for the renal arteries was more acute (LRA 148.4 ± 22.5°; RRA 140.0 ± 23.4°; CTR 154.1 ± 15.8°; SMA 156.5 ± 21.6°; *P* = 0.3) even if statistical significance was not reached. Moreover, as reported in Table 3, no significant differences were detected for any of the other computed parameters.

**Fig. 2:**
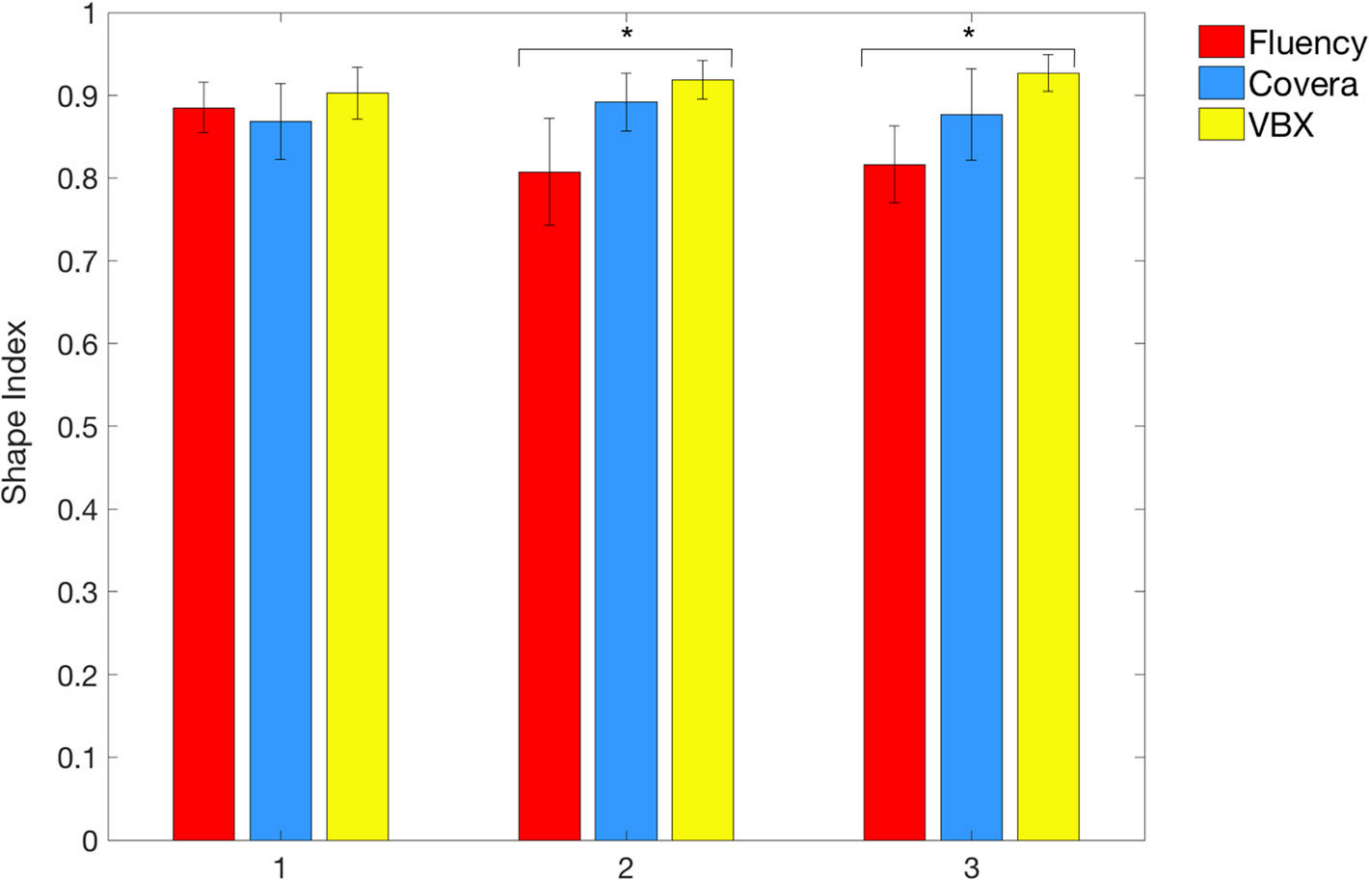
Bar plot showing differences in shape index between stent types in zones 1, 2, and 3 with significance of the different entries (“*” for *p* < 0.05). Error bars denote standard deviation

**Fig. 3:**
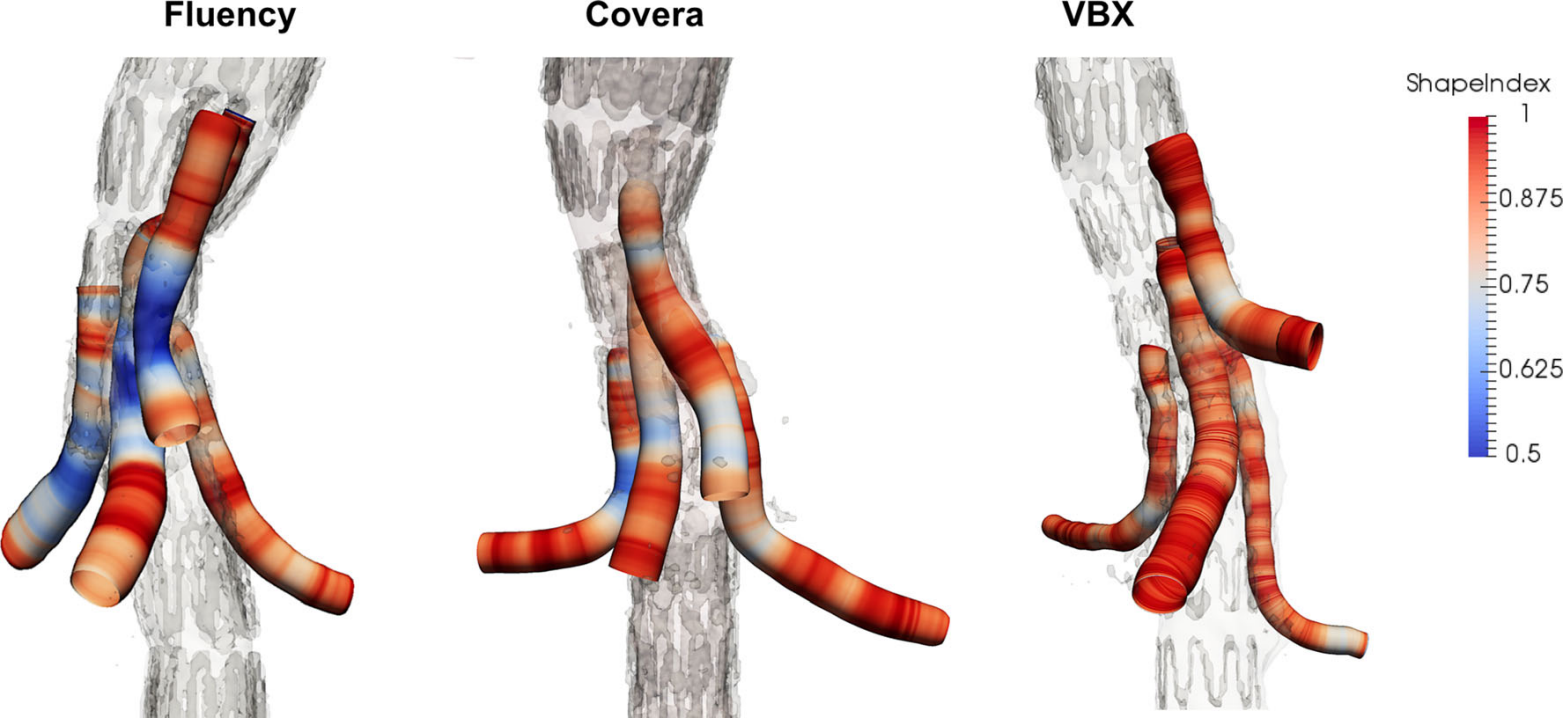
Shape index was computed for each section along the centerline path. Comparative outcomes concerning the three stent types (Fluency, Covera, and VBX) are reported. Color scale ranges from blue (minimum value; SI = 0.5) to red (maximum value; SI = 1). As highlighted, for the three comparative cases under consideration, better results are obtained for VBX stent

Grouping by bridging stent type, post-operative TOA was significantly more acute in the VBX group (140.6 ± 26.2°) in comparison to the Fluency (156.75 ± 13.74°) and Covera (158.2 ± 11.7°) (*P* = 0.05). Bar plot concerning SI average values on stent zones 1, 2, and 3 are reported in Fig. 2. Comparable values among the three stent types were found in zone 1 (*P* = 0.08), whereas a significantly higher average SI in VBX group was detected in zones 2 (*P* < 0.001) and 3 (*P* < 0.001). No significant differences in nominal stent length were found among the stent groups (*P* = 0.5). Exemplificative outcomes of the three stent types concerning shape index computation are given in Fig. 3.

Longitudinal stent shortening was computed considering only a single stent (*n* = 34). The VBX group resulted as the most affected by percentage shortening (11.12 ± 5.65%) in comparison to Fluency and Covera (4.86 ± 6.63% and 2.23 ± 2.68%, respectively) (*P* = 0.001). No statistically significant correlation was found between the nominal stent diameter and the longitudinal stent shortening variables.

### Intraobserver and Interobserver Reproducibility

Intraobserver and interobserver reproducibility were computed for manual measurements by ICC. All measurements showed excellent agreement both for intraobserver (ICC > 0.955) and interobserver (ICC > 0.935) variability.

## Discussion

A retrospective geometric analysis that takes into account both information on the angulation of the vessel and on the specific behavior of the stent once implanted was carried out with the final goal of the study being the investigation of bridging stent performances aiming to identify a stent with the best characteristics to be considered as ideal.

Recently, De Niet et al. [15] conducted a geometric investigation demonstrating that the anatomical configuration of branches in B/F-EVAR changes over time by measuring the most prominent angle along the branch centerline. In our study, we focused on the measurement of the TOA measured at native vessel origin. Pre-operative analysis confirmed that renal arteries have a more acute TOA if compared to celiac trunk and superior mesenteric arteries. The greater angulation of these vessels, together with a lower aortic diameter at the pararenal aorta if compared with the suprarenal aorta, makes these vessels the most at risk for complications, as already postulated by clinical investigation of Mastracci et al. [16] and Martin-Gonzales et al. [7]. In particular, they observed that renal arteries downward angulation following B-EVAR is associated with higher stress values in correspondence of native vessel origin which in turn are correlated with endothelial damage and increased local thrombogenic activity possibly leading to thrombosis. Interestingly, in our case series, it has been noted that in the post-operative configuration the TOA has undergone a smaller increase in angle, and therefore a lower vessel straightening in the VBX group if compared with the other stent groups.

As postulated by Mendes and Oderich [12], an ideal bridging stent should have a structure with adaptable characteristics depending on the segment being considered. The most proximal segment should be equipped with a high radial force to ensure stability over time in the area of overlap with the branch of the main body. The intermediate portion should be more flexible but also ensure a high resistance to kinking, while the more distal portion should be flexible and with high radial force in order to exert a good sealing in case of high-calcified target vessels. Such features would allow the bridging stent to adapt optimally to different anatomies.

In the present study, we hypothesized that radial force, rather than improved conformability is a key feature in the treatment of TAAAs with B-EVAR. With this in mind, we performed a geometric analysis characterizing the three stented segments with segmental centerline length and section SI, a parameter which evaluates the adaptability of the stent along its whole length.

As expected, comparable results in terms of SI between stent groups were found in zone 1 since it was always the branch of the main prosthesis that guided the shape in this section.

As concerns the stent behavior in the intermediate zone (zone 2), we observed a greater circularity of the stent along its length in the VBX stent group. In this portion, the stent was inside the aneurysmal sac, which, we believe, is the most critical area. In fact, if the stent does not have enough radial force, it is more likely to angle and create localized kinking zones.

Finally, as regards zone 3, VBX performs better in terms of adaptability within the target vessel. The explanation could be related to the intrinsic nature and material of the device. Shape index is more constant along the stent without localized kinking zones. In contrast, it has been shown that the VBX is subject to a greater shortening inside the target vessel that pays to maintain its circularity. This results in a shorter length L3 and hence a greater instability of the stent. This outcome correlates with the clinical evaluations already reported by Tenorio et al.[4] who recently analyzed the results of the treatment using the VBX. In particular, they observed a higher target artery instability rate in the case of balloon-expandable stents (VBX) compared to self-expanding ones with type IC endoleak, defined as endoleak originating from the distal target vessel sealing zone[17], being the main cause of reintervention. In our experience, even with limited follow-up, one patient had to undergo reintervention due to type IC endoleak.

New-generation VBX stent outcomes have been also compared with the Advanta V12/iCast (Getinge Maquet, Rastatt) in F-EVAR and B-EVAR procedures[18]. Authors hypothesized an improved trackability and flexibility of VBX device which in turn was responsible for 75% of type IC endoleaks detected during follow-up.

For this reason, better pre-operative planning when choosing the device length is imperative, taking into account that once implanted, there is a percentage shortening of the stent of about 10% of its nominal length, irrespective to the nominal stent diameter. One of the possible solutions to this problem could be to extend the range of lengths availability so as to have a stent that achieves the desired measures.

The other important aspect is to quantify the overlap of the bridging stent with the target vessel: little overlap can, over time, lead to a displacement of the stent and therefore to endoleak occurrence. Compatibly with the anatomy of the vessel to be treated and the collateral branches, a sealing zone in the target vessel of at least 2 cm should be created in order to ensure an appropriate sealing even with stent shortening.

The limited number of patients and the lack of long-term follow-up represent potentially relevant limitations. However, it should be pointed out that this is a preliminary investigation mainly focusing on geometrical analysis methodologies and that the conclusions we draw are not influenced by the limited number of patients. Furthermore, in the present study the results were not evaluated also considering the different types of B-EVAR graft adopted. In a subsequent study with a greater number of patients, we propose to consider this variable as well. Finally, future studies with a greater number of patients will also provide for the analysis of the study outcomes grouped both by type of stent and by target vessel.

## Conclusions

Despite the limitations of this study, our results suggest that the latest generation bridging stents can satisfactorily adapt to aortic anatomies, but some technical precautions must be taken into consideration to improve the results and therefore reduce the need for reoperations during follow-up. Geometrical outcomes of our study seem to suggest that the VBX offers better adaptability to varying anatomies. Particular attention, however, must be paid concerning the adequate overlap of the bridging stent inside the target vessel.
